# 2-(4-Chloro­phen­yl)-2-oxoethyl 3,4-dimeth­oxy­benzoate

**DOI:** 10.1107/S1600536811048264

**Published:** 2011-11-19

**Authors:** Hoong-Kun Fun, Ching Kheng Quah, A. M. Vijesh, A. M. Isloor, T. Arulmoli

**Affiliations:** a X-ray Crystallography Unit, School of Physics, Universiti Sains Malaysia, 11800 USM, Penang, Malaysia; b SeQuent Scientific Ltd, No. 120 A & B, Industrial Area, Baikampady, New Mangalore, Karnataka 575 011, India; c Medicinal Chemistry Division, Department of Chemistry, National Institute of Technology-Karnataka, Surathkal, Mangalore 575 025, India

## Abstract

In the title compound, C_17_H_15_ClO_5_, the benzene rings forms a dihedral angle of 74.45 (10)°. In the crystal, mol­ecules are linked into *C*(13) chains along [011] *via* C—H⋯O hydrogen bonds. The crystal packing also features short Cl⋯Cl contacts of 3.1253 (10) Å.

## Related literature

For a related structure and background to the properties and applications of phenacyl benzoate derivatives, see: Fun *et al.* (2011 ▶). For reference bond-length data, see: Allen *et al.* (1987 ▶).
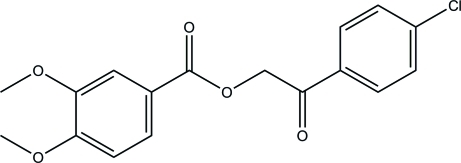


## Experimental

### 

#### Crystal data

C_17_H_15_ClO_5_
*M* *_r_* = 334.74
Triclinic, 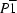

*a* = 8.2277 (6) Å
*b* = 9.3380 (6) Å
*c* = 10.5986 (7) Å
α = 89.062 (2)°
β = 76.752 (2)°
γ = 83.674 (2)°
*V* = 787.76 (9) Å^3^
*Z* = 2
Mo *K*α radiation
μ = 0.27 mm^−1^
*T* = 296 K
0.31 × 0.22 × 0.13 mm

#### Data collection

Bruker SMART APEXII DUO CCD diffractometer
Absorption correction: multi-scan (*SADABS*; Bruker, 2009 ▶) *T*_min_ = 0.922, *T*_max_ = 0.966
12242 measured reflections
4535 independent reflections
3056 reflections with *I* > 2σ(*I*)
*R*_int_ = 0.022

#### Refinement

*R*[*F* ^2^ > 2σ(*F* ^2^)] = 0.052
*wR*(*F* ^2^) = 0.234
*S* = 1.05
4535 reflections
210 parameters
H-atom parameters constrained
Δρ_max_ = 0.34 e Å^−3^
Δρ_min_ = −0.44 e Å^−3^

### 

Data collection: *APEX2* (Bruker, 2009 ▶); cell refinement: *SAINT* (Bruker, 2009 ▶); data reduction: *SAINT*; program(s) used to solve structure: *SHELXTL* (Sheldrick, 2008 ▶); program(s) used to refine structure: *SHELXTL*; molecular graphics: *SHELXTL*; software used to prepare material for publication: *SHELXTL* and *PLATON* (Spek, 2009 ▶).

## Supplementary Material

Crystal structure: contains datablock(s) global, I. DOI: 10.1107/S1600536811048264/hb6500sup1.cif

Structure factors: contains datablock(s) I. DOI: 10.1107/S1600536811048264/hb6500Isup2.hkl

Supplementary material file. DOI: 10.1107/S1600536811048264/hb6500Isup3.cml

Additional supplementary materials:  crystallographic information; 3D view; checkCIF report

## Figures and Tables

**Table 1:** Hydrogen-bond geometry (Å, °)

*D*—H⋯*A*	*D*—H	H⋯*A*	*D*⋯*A*	*D*—H⋯*A*
C2—H2*A*⋯O5^i^	0.93	2.58	3.397 (3)	147

Symmetry code: (i) 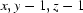.

**Fig. 1.**
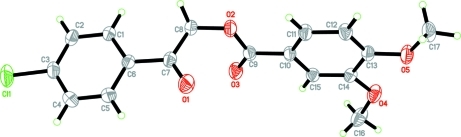
The molecular structure of the title compound showing 30% probability displacement ellipsoids for non-H atoms.

**Fig. 2.**
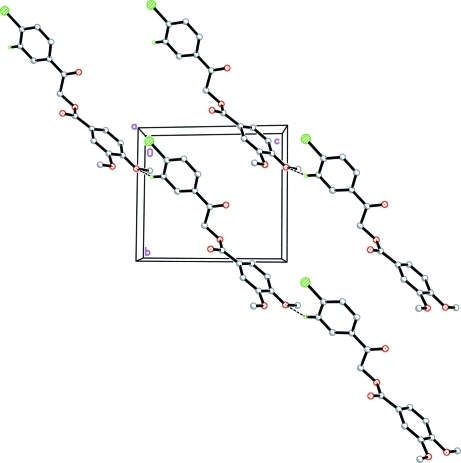
The crystal structure of the title compound, viewed along the a axis. H atoms not involved in hydrogen bonds (dashed lines) have been omitted for clarity.

## supplementary crystallographic information

### Comment

As part of our ongoing studies of phenacyl benzoates (Fun *et al.*, 2011) with possible applications in medicinal chemistry, we hereby report the crystal structure of the title compound, (I).

The molecular structure of the title compound is shown in Fig. 1. The chloro-bound phenyl (C1-C6) and benzene (C10-C15) rings form a dihedral angle of 74.45 (10) °. Bond lengths (Allen *et al.*, 1987) and angles are within normal ranges and are comparable to a related structure (Fun *et al.*, 2011).

In the crystal (Fig. 2), the molecules are linked into one-dimensional chains along [011] *via* intermolecular C2–H2A···O1^i^ hydrogen bonds (Table 1). There is a short Cl1···Cl1 contact (symmetry code: 1-x, -y, -z) with distance = 3.1253 (10) Å which is shorter than the sum of van der Waals radii of the clorine atoms.

### Experimental

The mixture of 3,4-dimethoxybenzoic acid (1.0 g, 0.0055 mol) potassium carbonate (0.95 g, 0.0069 mol) and 2-bromo-1-(4-chlorophenyl)ethanone (1.28 g, 0.0055 mol) in dimethyl formamide (10 ml) was stirred at room temperature for 2 h. On cooling, colorless needles of (I) begins to separate. They were collected by filtration and recrystallized from ethanol. Yield: 1.59 g, 86.9 %, *m.p.*: 415-416 K.

### Refinement

All H atoms were positioned geometrically and refined using a riding model with C–H = 0.93-0.98 Å and *U*_iso_(H) = 1.2 or 1.5 *U*_eq_(C). A rotating-group model was applied for the methyl groups.

### Crystal data

**Table:**

C_17_H_15_ClO_5_	*Z* = 2
*M**_r_* = 334.74	*F*(000) = 348
Triclinic, *P*1	*D*_x_ = 1.411 Mg m^−^^3^
Hall symbol: -P 1	Mo *K*α radiation, λ = 0.71073 Å
*a* = 8.2277 (6) Å	Cell parameters from 3650 reflections
*b* = 9.3380 (6) Å	θ = 2.6–29.9°
*c* = 10.5986 (7) Å	µ = 0.27 mm^−^^1^
α = 89.062 (2)°	*T* = 296 K
β = 76.752 (2)°	Needle, colourless
γ = 83.674 (2)°	0.31 × 0.22 × 0.13 mm
*V* = 787.76 (9) Å^3^	

### Data collection

**Table:**

Bruker SMART APEXII DUO CCD diffractometer	4535 independent reflections
Radiation source: fine-focus sealed tube	3056 reflections with *I* > 2σ(*I*)
graphite	*R*_int_ = 0.022
φ and ω scans	θ_max_ = 30.1°, θ_min_ = 2.0°
Absorption correction: multi-scan (*SADABS*; Bruker, 2009)	*h* = −11→11
*T*_min_ = 0.922, *T*_max_ = 0.966	*k* = −13→11
12242 measured reflections	*l* = −14→14

### Refinement

**Table:**

Refinement on *F*^2^	Primary atom site location: structure-invariant direct methods
Least-squares matrix: full	Secondary atom site location: difference Fourier map
*R*[*F*^2^ > 2σ(*F*^2^)] = 0.052	Hydrogen site location: inferred from neighbouring sites
*wR*(*F*^2^) = 0.234	H-atom parameters constrained
*S* = 1.05	*w* = 1/[σ^2^(*F*_o_^2^) + (0.1525*P*)^2^ + 0.0576*P*] where *P* = (*F*_o_^2^ + 2*F*_c_^2^)/3
4535 reflections	(Δ/σ)_max_ = 0.001
210 parameters	Δρ_max_ = 0.34 e Å^−^^3^
0 restraints	Δρ_min_ = −0.44 e Å^−^^3^

### Special details

**Table:**

Geometry. All esds (except the esd in the dihedral angle between two l.s. planes) are estimated using the full covariance matrix. The cell esds are taken into account individually in the estimation of esds in distances, angles and torsion angles; correlations between esds in cell parameters are only used when they are defined by crystal symmetry. An approximate (isotropic) treatment of cell esds is used for estimating esds involving l.s. planes.
Refinement. Refinement of F^2^ against ALL reflections. The weighted R-factor wR and goodness of fit S are based on F^2^, conventional R-factors R are based on F, with F set to zero for negative F^2^. The threshold expression of F^2^ > 2sigma(F^2^) is used only for calculating R-factors(gt) etc. and is not relevant to the choice of reflections for refinement. R-factors based on F^2^ are statistically about twice as large as those based on F, and R- factors based on ALL data will be even larger.

### Fractional atomic coordinates and isotropic or equivalent isotropic displacement parameters (Å^2^)

**Table:**

	*x*	*y*	*z*	*U*_iso_*/*U*_eq_	
Cl1	0.43412 (9)	0.12795 (7)	0.09809 (7)	0.0826 (3)	
O1	0.2269 (3)	0.5869 (2)	0.59869 (14)	0.0726 (5)	
O2	0.05337 (18)	0.83733 (16)	0.56180 (15)	0.0570 (4)	
O3	0.31494 (19)	0.90247 (18)	0.50295 (14)	0.0618 (4)	
O4	0.2833 (2)	1.30026 (19)	0.84644 (15)	0.0648 (5)	
O5	−0.0100 (2)	1.30829 (18)	0.99126 (14)	0.0638 (4)	
C1	0.1905 (3)	0.4855 (2)	0.27869 (18)	0.0484 (4)	
H1A	0.1148	0.5615	0.2632	0.058*	
C2	0.2470 (3)	0.3768 (2)	0.1871 (2)	0.0543 (5)	
H2A	0.2108	0.3791	0.1101	0.065*	
C3	0.3586 (3)	0.2648 (2)	0.2128 (2)	0.0530 (5)	
C4	0.4143 (3)	0.2581 (3)	0.3249 (3)	0.0674 (6)	
H4A	0.4896	0.1814	0.3397	0.081*	
C5	0.3572 (3)	0.3669 (2)	0.4157 (2)	0.0593 (5)	
H5A	0.3938	0.3631	0.4926	0.071*	
C6	0.2449 (2)	0.48282 (19)	0.39351 (16)	0.0418 (4)	
C7	0.1915 (2)	0.5993 (2)	0.49354 (17)	0.0467 (4)	
C8	0.0964 (3)	0.7348 (2)	0.4583 (2)	0.0510 (5)	
H8A	0.1644	0.7770	0.3828	0.061*	
H8B	−0.0054	0.7113	0.4358	0.061*	
C9	0.1789 (2)	0.9134 (2)	0.57600 (18)	0.0467 (4)	
C10	0.1251 (2)	1.01115 (19)	0.69027 (17)	0.0443 (4)	
C11	−0.0310 (3)	1.0126 (2)	0.7725 (2)	0.0510 (5)	
H11A	−0.1037	0.9479	0.7590	0.061*	
C12	−0.0813 (3)	1.1103 (2)	0.87599 (19)	0.0508 (5)	
H12A	−0.1872	1.1106	0.9310	0.061*	
C13	0.0261 (2)	1.2065 (2)	0.89671 (17)	0.0457 (4)	
C14	0.1885 (2)	1.2027 (2)	0.81470 (17)	0.0449 (4)	
C15	0.2367 (2)	1.1067 (2)	0.71224 (17)	0.0433 (4)	
H15A	0.3430	1.1050	0.6576	0.052*	
C16	0.4543 (3)	1.2941 (4)	0.7765 (2)	0.0756 (8)	
H16A	0.5112	1.3597	0.8151	0.113*	
H16B	0.4584	1.3204	0.6880	0.113*	
H16C	0.5082	1.1979	0.7794	0.113*	
C17	−0.1706 (3)	1.3195 (3)	1.0768 (2)	0.0704 (7)	
H17A	−0.1783	1.3946	1.1394	0.106*	
H17B	−0.1871	1.2296	1.1207	0.106*	
H17C	−0.2554	1.3418	1.0284	0.106*	

### Atomic displacement parameters (Å^2^)

**Table:**

	*U*^11^	*U*^22^	*U*^33^	*U*^12^	*U*^13^	*U*^23^
Cl1	0.0809 (5)	0.0657 (4)	0.0905 (5)	−0.0085 (3)	0.0055 (3)	−0.0403 (3)
O1	0.0967 (13)	0.0770 (11)	0.0437 (8)	0.0070 (9)	−0.0223 (8)	−0.0125 (7)
O2	0.0492 (8)	0.0534 (8)	0.0659 (9)	−0.0067 (6)	−0.0058 (6)	−0.0238 (7)
O3	0.0507 (8)	0.0716 (10)	0.0580 (8)	−0.0082 (7)	0.0002 (6)	−0.0221 (7)
O4	0.0603 (9)	0.0731 (10)	0.0626 (9)	−0.0264 (8)	−0.0069 (7)	−0.0200 (8)
O5	0.0633 (9)	0.0717 (10)	0.0532 (8)	−0.0114 (7)	−0.0028 (7)	−0.0268 (7)
C1	0.0541 (11)	0.0444 (9)	0.0500 (10)	−0.0020 (7)	−0.0197 (8)	−0.0070 (7)
C2	0.0626 (12)	0.0537 (11)	0.0502 (10)	−0.0094 (9)	−0.0176 (9)	−0.0120 (8)
C3	0.0481 (10)	0.0473 (10)	0.0599 (11)	−0.0090 (8)	−0.0021 (8)	−0.0159 (8)
C4	0.0667 (14)	0.0561 (12)	0.0789 (15)	0.0152 (10)	−0.0252 (12)	−0.0127 (11)
C5	0.0662 (13)	0.0572 (12)	0.0579 (11)	0.0074 (10)	−0.0273 (10)	−0.0083 (9)
C6	0.0432 (9)	0.0416 (9)	0.0418 (8)	−0.0070 (7)	−0.0106 (7)	−0.0033 (7)
C7	0.0491 (10)	0.0497 (10)	0.0411 (8)	−0.0078 (8)	−0.0081 (7)	−0.0074 (7)
C8	0.0501 (10)	0.0487 (10)	0.0545 (10)	−0.0020 (8)	−0.0133 (8)	−0.0156 (8)
C9	0.0438 (9)	0.0447 (9)	0.0496 (9)	−0.0032 (7)	−0.0069 (7)	−0.0080 (7)
C10	0.0456 (9)	0.0411 (9)	0.0446 (9)	−0.0032 (7)	−0.0069 (7)	−0.0079 (7)
C11	0.0482 (10)	0.0498 (10)	0.0532 (10)	−0.0107 (8)	−0.0048 (8)	−0.0099 (8)
C12	0.0463 (10)	0.0543 (11)	0.0474 (9)	−0.0078 (8)	0.0002 (8)	−0.0091 (8)
C13	0.0518 (10)	0.0455 (9)	0.0388 (8)	−0.0035 (7)	−0.0088 (7)	−0.0062 (7)
C14	0.0476 (9)	0.0455 (9)	0.0437 (9)	−0.0094 (7)	−0.0123 (7)	−0.0031 (7)
C15	0.0408 (9)	0.0452 (9)	0.0424 (8)	−0.0048 (7)	−0.0061 (7)	−0.0021 (7)
C16	0.0647 (14)	0.107 (2)	0.0586 (12)	−0.0436 (14)	−0.0054 (10)	−0.0113 (13)
C17	0.0721 (15)	0.0784 (16)	0.0519 (12)	0.0014 (12)	0.0013 (10)	−0.0237 (11)

### Geometric parameters (Å, °)

**Table:**

Cl1—C3	1.739 (2)	C7—C8	1.502 (3)
O1—C7	1.215 (2)	C8—H8A	0.9700
O2—C9	1.353 (2)	C8—H8B	0.9700
O2—C8	1.423 (2)	C9—C10	1.482 (2)
O3—C9	1.201 (2)	C10—C11	1.376 (3)
O4—C14	1.356 (2)	C10—C15	1.407 (2)
O4—C16	1.427 (3)	C11—C12	1.396 (3)
O5—C13	1.352 (2)	C11—H11A	0.9300
O5—C17	1.415 (3)	C12—C13	1.381 (3)
C1—C2	1.382 (3)	C12—H12A	0.9300
C1—C6	1.389 (3)	C13—C14	1.414 (3)
C1—H1A	0.9300	C14—C15	1.377 (2)
C2—C3	1.380 (3)	C15—H15A	0.9300
C2—H2A	0.9300	C16—H16A	0.9600
C3—C4	1.367 (3)	C16—H16B	0.9600
C4—C5	1.378 (3)	C16—H16C	0.9600
C4—H4A	0.9300	C17—H17A	0.9600
C5—C6	1.397 (3)	C17—H17B	0.9600
C5—H5A	0.9300	C17—H17C	0.9600
C6—C7	1.489 (2)		
			
C9—O2—C8	115.33 (15)	O2—C9—C10	111.37 (16)
C14—O4—C16	117.59 (17)	C11—C10—C15	119.80 (16)
C13—O5—C17	118.36 (17)	C11—C10—C9	121.82 (16)
C2—C1—C6	121.01 (18)	C15—C10—C9	118.36 (16)
C2—C1—H1A	119.5	C10—C11—C12	120.61 (17)
C6—C1—H1A	119.5	C10—C11—H11A	119.7
C3—C2—C1	118.36 (18)	C12—C11—H11A	119.7
C3—C2—H2A	120.8	C13—C12—C11	119.99 (17)
C1—C2—H2A	120.8	C13—C12—H12A	120.0
C4—C3—C2	122.28 (19)	C11—C12—H12A	120.0
C4—C3—Cl1	118.43 (18)	O5—C13—C12	125.37 (18)
C2—C3—Cl1	119.28 (17)	O5—C13—C14	115.02 (16)
C3—C4—C5	119.0 (2)	C12—C13—C14	119.61 (16)
C3—C4—H4A	120.5	O4—C14—C15	125.87 (17)
C5—C4—H4A	120.5	O4—C14—C13	114.09 (16)
C4—C5—C6	120.66 (19)	C15—C14—C13	120.04 (16)
C4—C5—H5A	119.7	C14—C15—C10	119.91 (16)
C6—C5—H5A	119.7	C14—C15—H15A	120.0
C1—C6—C5	118.69 (18)	C10—C15—H15A	120.0
C1—C6—C7	123.08 (17)	O4—C16—H16A	109.5
C5—C6—C7	118.22 (16)	O4—C16—H16B	109.5
O1—C7—C6	121.47 (18)	H16A—C16—H16B	109.5
O1—C7—C8	121.02 (18)	O4—C16—H16C	109.5
C6—C7—C8	117.49 (15)	H16A—C16—H16C	109.5
O2—C8—C7	111.92 (16)	H16B—C16—H16C	109.5
O2—C8—H8A	109.2	O5—C17—H17A	109.5
C7—C8—H8A	109.2	O5—C17—H17B	109.5
O2—C8—H8B	109.2	H17A—C17—H17B	109.5
C7—C8—H8B	109.2	O5—C17—H17C	109.5
H8A—C8—H8B	107.9	H17A—C17—H17C	109.5
O3—C9—O2	123.14 (17)	H17B—C17—H17C	109.5
O3—C9—C10	125.48 (17)		
			
C6—C1—C2—C3	0.3 (3)	O2—C9—C10—C11	3.8 (3)
C1—C2—C3—C4	0.0 (3)	O3—C9—C10—C15	4.4 (3)
C1—C2—C3—Cl1	−179.06 (15)	O2—C9—C10—C15	−174.77 (16)
C2—C3—C4—C5	0.0 (4)	C15—C10—C11—C12	1.5 (3)
Cl1—C3—C4—C5	179.11 (19)	C9—C10—C11—C12	−177.06 (18)
C3—C4—C5—C6	−0.4 (4)	C10—C11—C12—C13	−0.1 (3)
C2—C1—C6—C5	−0.7 (3)	C17—O5—C13—C12	−0.3 (3)
C2—C1—C6—C7	178.23 (17)	C17—O5—C13—C14	179.6 (2)
C4—C5—C6—C1	0.7 (3)	C11—C12—C13—O5	178.19 (19)
C4—C5—C6—C7	−178.2 (2)	C11—C12—C13—C14	−1.7 (3)
C1—C6—C7—O1	172.1 (2)	C16—O4—C14—C15	−6.9 (3)
C5—C6—C7—O1	−8.9 (3)	C16—O4—C14—C13	174.0 (2)
C1—C6—C7—C8	−9.6 (3)	O5—C13—C14—O4	1.4 (3)
C5—C6—C7—C8	169.30 (19)	C12—C13—C14—O4	−178.68 (19)
C9—O2—C8—C7	79.1 (2)	O5—C13—C14—C15	−177.72 (17)
O1—C7—C8—O2	−1.5 (3)	C12—C13—C14—C15	2.2 (3)
C6—C7—C8—O2	−179.80 (15)	O4—C14—C15—C10	−179.87 (18)
C8—O2—C9—O3	4.0 (3)	C13—C14—C15—C10	−0.8 (3)
C8—O2—C9—C10	−176.80 (16)	C11—C10—C15—C14	−1.0 (3)
O3—C9—C10—C11	−177.1 (2)	C9—C10—C15—C14	177.60 (16)

### Hydrogen-bond geometry (Å, °)

**Table:**

*D*—H···*A*	*D*—H	H···*A*	*D*···*A*	*D*—H···*A*
C2—H2A···O5^i^	0.93	2.58	3.397 (3)	147

Symmetry codes: (i) *x*, *y*−1, *z*−1.
